# Thalidomide against Coronavirus Disease 2019 (COVID-19): A Medicine with a Thousand Faces

**DOI:** 10.22037/ijpr.2020.113369.14259

**Published:** 2020

**Authors:** Farzaneh Dastan, Payam Tabarsi, Majid Marjani, Afshin Moniri, Seyed Mohammad Reza Hashemian, Maria Tavakoli-Ardakani, Ali Saffaei

**Affiliations:** a *Department of Clinical Pharmacy, School of Pharmacy, Shahid Beheshti University of Medical Sciences, Tehran, Iran.*; b *Chronic Respiratory Diseases Research Center, National Research Institute of Tuberculosis and Lung Diseases (NRITLD), Shahid Beheshti University of Medical Sciences, Tehran, Iran. *; c *Clinical Tuberculosis and Epidemiology Research Center, National Research Institute of Tuberculosis and Lung Diseases (NRITLD), Masih Daneshvari Hospital, Shahid Beheshti University of Medical Sciences, Tehran, Iran. *; d *Virology Research Center, National Research Institute of Tuberculosis and Lung Diseases (NRITLD), Masih Daneshvari Hospital, Shahid Beheshti University of Medical Sciences, Tehran, Iran. *; e *Student Research Committee, Department of Clinical Pharmacy, School of Pharmacy, Shahid Beheshti University of Medical Sciences, Tehran, Iran.*

**Dear Editor**

Recently, Wuhan, China became the epicenter for the outbreak of novel coronavirus pneumonia (COVID-19), which is associated with multiorgan failure, including acute respiratory distress syndrome (ARDS), acute cardiac injury, and shock (1, 2). Currently, no specific agents are available for treating COVID-19 while some agents such as antivirals, chloroquine, and immunomodulatory agents are under investigation (3). Among them, thalidomide is a promising candidate agent.

Nearly 60 years ago, thalidomide was recommended to manage morning sickness in pregnancy. After years, the biggest medical disaster occurred, where nearly 10,000 children were born with severe malformations (4). Despite this disaster, thalidomide is now used effectively to treat a range of diseases. Modulating cytokines is among the wide range of biological activities thalidomide possesses. Previous studies have evaluated its anxiolytic, sedative-hypnotic, antiemetic, and analgesic effects. Consequently, thalidomide proved beneficial in Hansen’s disease, myeloma, myelodysplastic syndrome, infectious diseases, autoimmune diseases, malignancy, and wasting syndrome related to malignancy and acquired immune deficiency syndrome (AIDS) (5). 

*In-vitro* and *in-vivo* studies showed that thalidomide impairs the synthesis of tumor necrosis factor alpha (TNF-alpha). It increases peripheral blood CD8+ T cells, plasma interleukin 12 (IL-12) levels, interferon-γ production, and cytotoxic activity. 

*In-vitro *study by Tabata *et al.* revealed thalidomide decrease the expression of IL-1β and IL-6 in human lung epithelial cells and may prove helpful in preventing emphysema (6). In an animal study by Dong *et al.*, thalidomide remarkably attenuated pulmonary fibrosis, oxidative stress, and inflammation in mice lungs (7). Amirshahrokhi *et al.* reported similar findings which showed thalidomide decreased production of TNF-alpha, IL-1β, IL-6, and transforming growth factor-β (8). Uthoff *et al.* also studied the effects of thalidomide in lung transplanted dogs. They observed thalidomide is better than corticosteroids in early postoperative immunosuppression after lung transplantation and that it is associated with a decreased incidence of pneumonia (9). Dong *et al.* also reported that thalidomide has anti-fibrotic effects against bleomycin-induced pulmonary fibrosis in rats (10). Anti-inflammatory effects of thalidomide on H1N1 influenza virus-induced pulmonary injury in mice showed that thalidomide greatly improves the survival rate, reduces the infiltration of inflammatory cells, cytokine (*e.g.,* IL-6, TNF-α), and chemokine (chemokine ligand 5, C-X-C motif chemokine 10) levels, and inhibits the activated p-NFκB p6 (11, 12).

A recently published case report described the beneficial effects of thalidomide (100 mg orally once daily) in combination with low-dose glucocorticoid (13). Given the beneficial effects of thalidomide, it is necessary to try it in COVID-19 cases as a therapeutic agent. It is recommended that large clinical trials be designed to find the efficacy and safety of thalidomide in COVID-19.
